# Bidirectional Modulation of Nociception by GlyT2^+^ Neurons in the Ventrolateral Periaqueductal Gray

**DOI:** 10.1523/ENEURO.0069-23.2023

**Published:** 2023-06-12

**Authors:** Neda Assareh, Caitlin Fenech, Rebecca Power, Mohammad N. Uddin, Yo Otsu, Karin R. Aubrey

**Affiliations:** 1 Pain Management Research, Kolling Institute, Royal North Shore Hospital Northern Sydney Local Health District and Faculty of Medicine and Health, University of Sydney, Sydney, New South Wales 2065, Australia; 2 Sydney Pain Consortium, Faculty of Medicine and Health, University of Sydney, Sydney, New South Wales 2006, Australia; 3 School of Medical Sciences, Faculty of Medicine and Health, University of Sydney, Sydney, New South Wales 2006, Australia

**Keywords:** chemogenetics, glycinergic neurotransmission, GlyT2::Cre mice, nociception, supraspinal glycine, vlPAG

## Abstract

The midbrain periaqueductal gray (PAG), particularly its ventrolateral column (vlPAG), is part of a key descending pathway that modulates nociception, fear and anxiety behaviors in both humans and rodents. It has been previously demonstrated that inhibitory GABAergic neurons within the vlPAG have a major role in this nociceptive modulation. However, the PAG contains a diverse range of neuronal subtypes and the contribution of different subtypes of inhibitory neurons to nociceptive control has not been investigated. Here, we employed a chemogenetic strategy in mice that express Cre recombinase under the promotor for the glycine transporter 2 (GlyT2::cre) to modulate a novel group of glycinergic neurons within the vlPAG and then investigate their role in nociceptive control. We show that activation of GlyT2-PAG neurons enhances cold and noxious heat responses and increases locomotor activity (LMA) in both male and female mice. In contrast, inhibition of GlyT2-PAG neurons reduced nociceptive responses, while locomotor behaviors were unaffected. Our findings demonstrate that GlyT2^+^ neurons in the vlPAG modulate nociception and suggest that strategies targeting GlyT2-PAG neurons could be used to design novel analgesic therapies.

## Significance Statement

Neuronal circuits are composed of diverse collections of cell types, each with a distinct set of synaptic connections that determine their role in specific functions. One challenge in neuropharmacology is to design drugs that interact with the brain circuits required to have the desired therapeutic effect and limit their activity at nearby circuits, thus reducing side effects. The current study shows that a genetically identified subpopulation of GlyT2^+^ neurons that are concentrated in the ventrolateral periaqueductal gray (vlPAG) can bidirectionally modulate nociceptive responses and alter locomotion behaviors in mice. These findings provided novel insights into the organization of the nociceptive circuitry of the PAG and identify GlyT2-PAG neurons as a potential target for analgesic drug design.

## Introduction

Responding appropriately to pain (or the possibility of pain) requires the integration of nociceptive inputs with motor, autonomic and affective brain circuits (Behbehani, 1995; Benarroch, 2012). A detailed understanding of how each subpopulation of neurons within these regions contributes to shaping different pain aspects is needed to enhance our understanding of the cells involved and to develop effective new therapies for pain.

The midbrain periaqueductal gray (PAG) has extensive efferent and afferent connections that help it coordinate behavioral responses to various stressors and threats, including pain (Cameron et al., 1995a,b). A wealth of tracing and neurochemical analyses have demonstrated that the PAG is functionally subdivided into four columns, dorsomedial (dm), dorsolateral (dl), lateral (l), and ventrolateral (vl), each characterized by distinct afferent and efferent connections, molecular profiles, and associated behavior (Brandão et al., 1982; Zhang et al., 1990; Fanselow, 1991; Carrive, 1993; Bandler and Shipley, 1994; K.A. Keay and Bandler, 2015; Vaughn et al., 2022). Electrical and chemical stimulation studies indicate that the vlPAG drives antinociception and is involved in passive behavioral responses (e.g., quiescence and immobility), whereas the dlPAG and lPAG columns are involved in antinociception coupled with active responses (e.g., flight and defensive behaviors; M. Morgan et al., 1998; K.A. Keay et al., 2001; M.M. Morgan and Clayton, 2005; K. Keay and Bandler, 2008). The ability of the vlPAG to modulate nociception is primarily mediated by a key descending pathway that projects through the rostroventromedial medulla to suppress nociceptive transmission in the dorsal horn of the spinal cord (Vaughan and Christie, 1997; Lau and Vaughan, 2014). This descending pathway is also an important target for both endogenous and exogenous opioids (Mohrland and Gebhart, 1980; Cheng et al., 1986; Bagley and Ingram, 2020). Thus, the vlPAG helps shape complex behavioral responses to stressors, contributes to nociceptive control and can drive profound analgesia.

The vlPAG is made up of a diverse array of cell types characterized by their genetic and neurochemical profiles, and more broadly whether they release inhibitory or excitatory neurotransmitters. The distinct nociceptive roles of excitatory and inhibitory signaling within the vlPAG have been investigated using microinjection of specific GABA- or glutamate receptor agonists and antagonists (Moreau and Fields, 1986; Depaulis et al., 1987; Sandkühler et al., 1989). More recently genetic technologies in combination with chemogenetics or optogenetics have examined the specialized roles of vlPAG neuronal cell types (Tovote et al., 2016; Capelli et al., 2017; Samineni et al., 2017, 2019; Hao et al., 2019; Yin et al., 2020; Yu et al., 2021). Collectively, these studies confirm that activating glutamatergic neurons, or suppressing inhibitory GABAergic neurons is antinociceptive, consistent with disinhibition of the descending pain modulatory pathway (Lau and Vaughan, 2014). However, inhibitory neurotransmission can also be mediated by glycinergic neurons (Zeilhofer et al., 2018), although if and how glycinergic neurons contribute to different vlPAG-mediated behaviors is unknown.

Here, we investigate the behavioral outcome of chemogenetically modulating a subpopulation of inhibitory vlPAG neurons identified by their expression of the SLC6A5 gene, encoding the glycine transporter 2 (GlyT2). GlyT2 is an established marker of inhibitory glycinergic neurons and is responsible for accumulating glycine into presynaptic terminals (Zafra et al., 1995; Rampon et al., 1996; Zeilhofer et al., 2005; Aubrey et al., 2007). GlyT2^+^ neurons are found throughout the mammalian spinal cord and hindbrain (brainstem, cerebellum, and limited populations in the thalamus), but absent from forebrain regions (Rampon et al., 1996; Zeilhofer et al., 2005; Miranda et al., 2022) and in the adult CNS, they often corelease glycine with GABA (Chéry and De Koninck, 1999; Nabekura et al., 2004; Rousseau et al., 2012; Otsu and Aubrey, 2022). While their role in spinal nociceptive circuits have been intensely investigated, little is known about the involvement of glycinergic neurons in supra-medullary brain regions such as the midbrain PAG.

Previous reports have reported that a minority of inhibitory neurons in the vlPAG express GlyT2 (GlyT2-PAG neurons; Rampon et al., 1996; Tanaka and Ezure, 2004; Zeilhofer et al., 2005), but their functional role has never been investigated. Given the importance of the vlPAG in nociceptive responses, we hypothesise that GlyT2-PAG neurons may form part of a PAG microcircuit involved in nociceptive processing.

## Materials and Methods

### Animals

All experiments were conducted in accordance with the Australian Code for the care and use of animals for scientific purposes. A total of 65 adult transgenic mice with Cre-recombinase expressed under the promoter of the glycine transporter, GlyT2 (GlyT2::Cre mice, a gift from H. U. Zeilhofer; Foster et al., 2015) were used in this study. Seventy percent of these animals are included in the analysis after removal of those with a misplaced or lack of AAV delivery. In addition, *n* = 5 cre-negative littermates were used in control experiments to confirm that there were no behavioral effects of AAV5-DIO-hM3Dq cre-independent leak (Botterill et al., 2021). All protocols were approved by the Institutional Animal Ethics committee (Protocol no. RESP 19/66). Animals were bred and housed in the Institute Animal Facility in ventilated cages with a maximum of four mice/cage. Animals were maintained on a 12/12 h light/dark cycle (23°C, 70% humidity) and were provided food and water *ad libitum*. Cages were enriched with a house igloo, tissues for nesting, and straws or paddle pop sticks on alternate weeks.

### Viral constructs and surgery

Stereotaxic injections were performed on adult mice (8–12 weeks; 22 ± 3 g) under anesthesia (1.5–3% isoflurane and 1 l/min O_2_) using a stereotactic apparatus (Kopf Instruments). Cre dependent expression of control or chemogenetic products was achieved using AAV5-hSyn-DIO-mCherry (8.4e12 vg/ml, Addgene #50459), AAV5-hSyn-DIO-hM3D(Gq)-mCherry (2.3e13 vg/ml, Addgene #44361) and AAV5-hSyn-DIO-hM4D(Gi)-mCherry (2.5e13 vg/ml, Addgene #44362). Vectors were a kind gift from Brian Roth (Addgene catalog #44362, #44361, #50459).

The following coordinates were used to target the vlPAG: bregma Anterior-Posterior (AP), −4.7 to −4.9 mm; Medial-Lateral (ML), ±0.3–0.4 mm; Dorsal-Ventral (DV), 2.7–2.9 mm (Samineni et al., 2017). Injections were made with a motorized microinjector (UMP3T-2 with SMARTouch, WPI) and 200 nl of the vector was infused at the speed of 100 nl/min. Then, the nanovolum needle (SEG Syringe, volume 1 nl, 0.63 mm OD, part #000500) was kept in place for 5 min, retracted 0.1 mm and left for a further 3 min before complete withdrawal. 6–0 black braided silk sutures and iodine sterilization were used to aid in wound healing, and pain relief was administered (buprenorphine 0.05–0.1 μg/g mice, s.c). Following surgery, the mice were individually housed until recovery from the procedure (max 2 d) before being returned to their home cage.

### Chemogenetic manipulation

**Figure 1.**
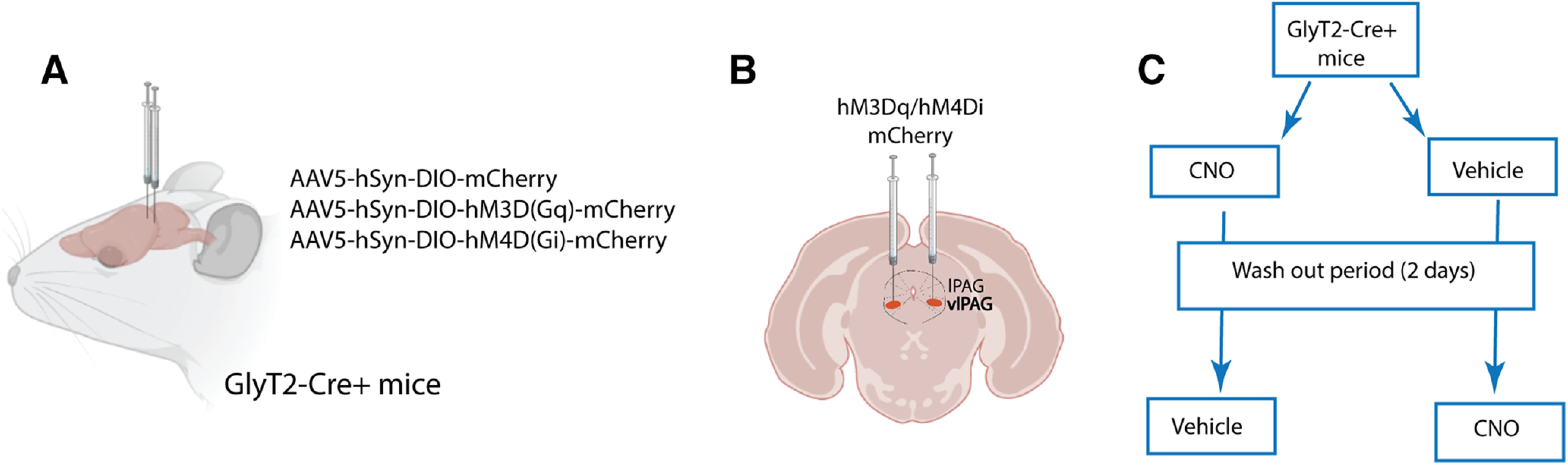
Experimental protocol. ***A***, ***B***, Viral vectors AAV5-hSyn-DIO-hM3Dq–mCherry, AAV5-hSyn-DIO-hM4Di–mCherry, and AAV5-hSyn-DIO-mCherry were bilaterally injected into the vlPAG of GlyT2::Cre mice three to four weeks before behavioral tests. ***C***, Behavioral schematic. CNO or vehicle injection (intraperitoneal) took place 1 h before behavioral testing. Test were conducted using a within-subjects crossover design and a 2-d rest period between CNO and vehicle to ensure complete CNO washout.

Three to four weeks after viral injection, mice underwent baseline behavioral testing and were then injected intraperitoneally with clozapine-N-oxide (CNO; diluted in 10% DMSO and saline, 3–5 mg/kg; Sigma-Aldrich) or vehicle control (10% DMSO and saline) and 60 min later behavioral experiments commenced. We used a within-subjects crossover design and animals had a 2-d rest period between CNO and vehicle treatments to ensure complete washout of CNO (Fig. 1). CNO was injected at 3 mg/kg and 5 mg/kg for animals expressing hM3D (Gq) activation and hM4D(Gi) inactivation vectors respectively as previously described (Alexander et al., 2009). Control animals expressing mCherry were administrated 5 mg/kg CNO.

Each animal underwent two (locomotor) or three (nociception) periods of testing following either: baseline (no treatment, nociceptive tests only), CNO or saline treatment. The baseline was determined before the first treatment and the order of CNO, and saline administration was counterbalanced. The researcher was blinded to the treatment group during the behavioral experiments and analysis.

We confirmed that AAV-Cre-independent leak of vectors (Botterill et al., 2021) does not contribute to the CNO-stimulated behavioral changes measured in our model as CNO (3 mg/kg, i.p) had no functional effect (acetone, hotplate, open field tests) in cre-negative littermates injected with AAV5-hSyn-DIO-hM3Dq–mCherry (*n* = 5; Extended Data Fig. 3-1).

### Nociceptive tests

Acute nociception was evaluated by measuring responses to cold (acetone) and hot (hotplate) stimuli applied to the left hind paw (>5 min between tests to ensure results are independent; Anderson et al., 2014; François et al., 2017; Samineni et al., 2017; Mitchell et al., 2021). For the acetone test (Yoon et al., 1994), mice were habituated to adjacent individual testing chambers with a wire-mesh floor for 30 min before testing started. The response to rapid cooling was measured by application of acetone (20 μl) to the plantar surface of the left hind paw with a pipette fitted with a customized tip. The number of licking responses was counted over 20 s and averaged over two trials (5 min between trials). For the hotplate test (Hunskaar et al., 1986; Vermeirsch and Meert, 2004), mice were individually placed in a cylindrical enclosure on a metal surface hotplate at 50°C (for a maximum of 45 s). The time taken to display a nociceptive response, such as licking or shaking of the hind paw, was averaged over three trials (5-min interval between trials). In mice where there was >4-s difference between the three trials, up to two extra trials were conducted and the average response was calculated from the three closest responses (Espejo et al., 1994).

### Locomotor activity

Locomotor activity (LMA) was assessed one week after the sensory testing using the open field test. Mice were placed in an enclosed open-top arena (50 × 50 × 50 cm) and an overhead camera recorded the behavior of the mice for 20 min (light level = 45 lux). This period allowed for the monitoring of locomotion and anxiety-like behaviors (Bailey and Crawley, 2009). The following characteristics of locomotion were scored; total distance moved, average speed, total time of im/mobility, total line crossing, and total time, distance in the center zone, rearing, and jumping.

### Perfusion and fixation

On the day of perfusion, all mice received a saline or CNO injection (intraperitoneally). Two hours later, mice were deeply anaesthetized with an overdose of Lethabarb (200 mg/kg), and depth of anesthesia was verified by a lack of righting response or paw withdrawal in response to a foot pinch. Then, they were transcardially perfused with 0.9% saline containing 72.5 mm NaNO_2_ and 3 IU/ml heparin (Sigma), followed by 4% paraformaldehyde (PFA) in 0.13 m PBS (pH 7.4). Brains were removed, postfixed, and refrigerated overnight in the same fixative solution (4% PFA, 4°C). The tissue was washed in PBS and cryoprotected in 30% sucrose in PBS (pH 7.4 at 4°C) for 2 d before being stored at −80°C until cryo-sectioning.

### Viral placement and mCherry/cFos immunohistochemistry

A cryostat (−20°C, Leica Microsystems; Leica 1080) was used to collect serial 40-μm coronal sections of the PAG. These sections were collected in a 1:4 series in 24-well plates as free-floating sections and preserved in 0.1 m phosphate buffer (PB) saline containing 0.1% sodium azide at 4°C. The PAG slices from one series were mounted onto gelatinized glass slides with ProLong mounting media (Thermo Fischer Scientific) and a glass coverslip, allowed to dry, and imaged to confirm stereotaxic AAV-vector placement by visualizing mCherry expression (without amplification).

In a second series of tissue from mice expressing hM3Dq who received either saline or CNO before perfusion, the colocalization of cFos and mCherry was measured by combining cFos DAB-immunohistochemistry (IHC) with immunofluorescent (IF) labeling of mCherry. Sections were washed in 1× Envision FLEX wash buffer (DAKO, catalog #K800721-2, pH 7.6) followed by 50% ethanol and 50% ethanol with 3% hydrogen peroxide in DAKO FLEX wash buffer for 30 min each. Then, sections were incubated in c-Fos (1:5000, New England Biolabs, catalog #2250S) and mCherry primary antibodies (1:1000, Abcam, catalog #ab205402) in DAKO wash buffer for two nights at 4°C. Following washing in DAKO wash buffer, sections were incubated with Envision^+^ system-HRP-labeled polymer anti-rabbit (DAKO, catalog #K400311-2) at room temperature (RT) for 2 h and peroxidase activity was revealed using liquid DAB^+^ (DAKO, catalog #K346811-2) for 10 min. The reaction was stopped by the addition milliQ water. Finally, the sections were incubated for 2 h at RT with the secondary antibody for mCherry (goat anti-chicken IgY H + L Alexa Fluor 568 in 1:500, Abcam catalog #Ab175477) in DAKO wash buffer with 10% goat serum and DAPI (1:2000). After a final wash period, slices were mounted on slides using Epredia Lab Vision PermaFluor aqueous mounting medium (Thermo Fisher Scientific, catalog #TA-030-FM).

### Fluorescent and light microscopy

**Figure 2.**
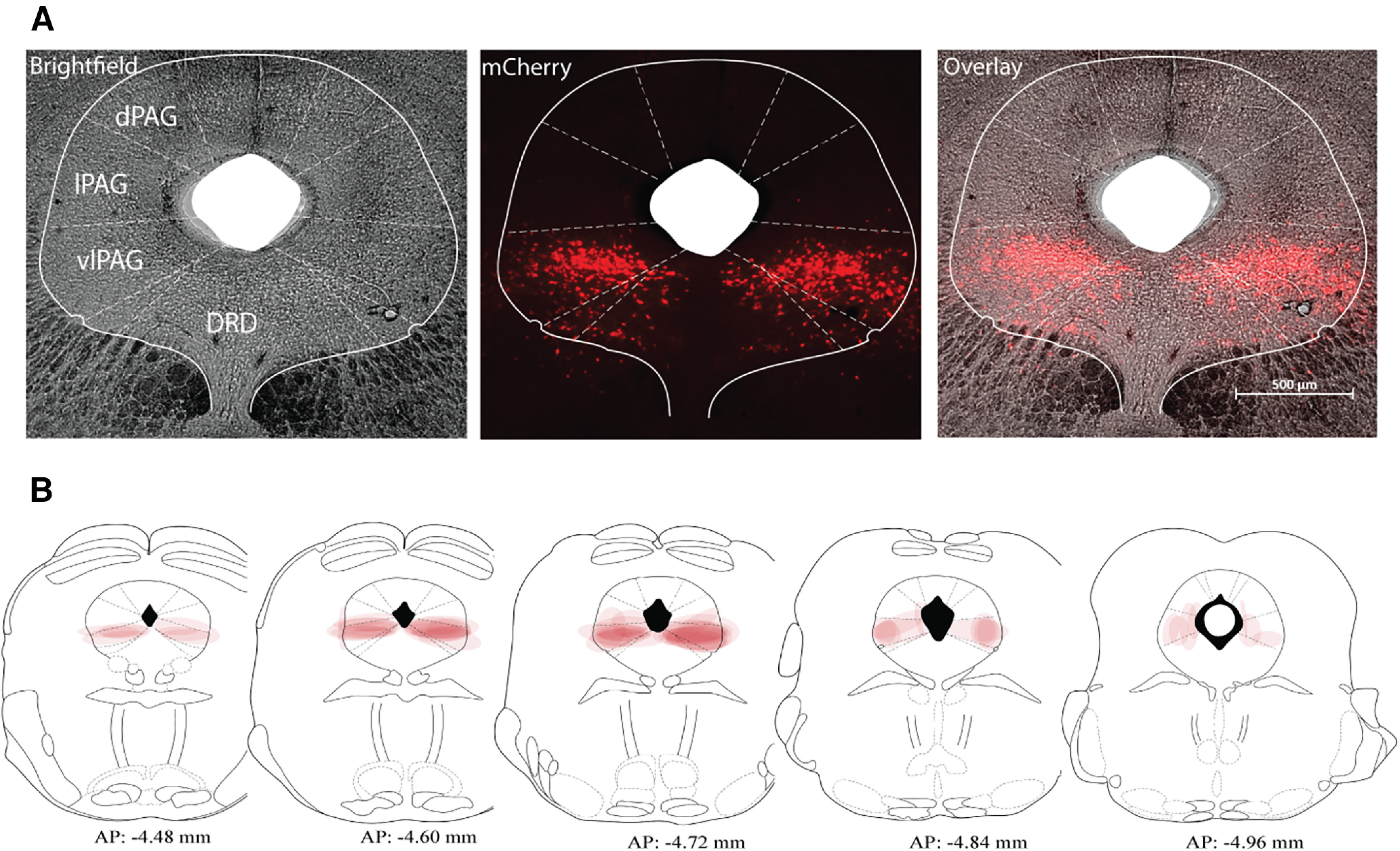
GlyT2-PAG neurons in the vlPAG expressed AAV-DREAADs-mCherry. ***A***, Representative microscopic images of coronal sections showing AAV5-hSyn-DIO-hM4D(Gi)-mCherry was largely restricted within the vlPAG: showing the expression of mCherry fluorescence (red), brightfield (gray), and overlayed of mCherry and brightfield images. Scale bars: 500 μm, Magnification: 2.5×. AP coordinate: −4.72 mm. ***B***, Placement map of AAV5 viral vector expression for all male mice included in the analyses in Figures 4, 5 showing 10% opacity for each animal.

PAG slices were imaged across the rostro-caudal axis on a fluorescent microscope (Zeiss Axio M1) and cFos was imaged using brightfield illumination. The bilateral localization of injection sites centered around the vlPAG was confirmed according to Paxinos and Franklin (2001). Mice with clear evidence of bilateral expression of the injected vector in the PAG were included in the analyses. To create Figures 2*B* and 6*A*, the highest level of viral expression per section was considered and schemed onto PAG templates. In a few animals, sparse labeling of neurons in the superior colliculus was noted near the injection tract.

### Electrophysiology

Four to five weeks after viral infection of PAG neurons, mice were deeply anesthetized with 2% isoflurane (assessed by the rate of breathing, lack of righting reflexes, and lack of response to paw squeeze) and transcardially perfused with ice-cold *N*-methyl-D-glucamine solution (NMDG) containing (in mm): 93 NMDG, 30 NaHCO3, 25 glucose, 5 *N*-acetyl-L-cysteine, 3 Na-pyruvate, 2.5 KCl, 1.2 NaH_2_PO_4_, 20 HEPES, 10 MgSO_4_, 0.5 CaCl_2_, 5 sodium ascorbate (∼300 mOsm), equilibrated with 95% O_2_-5% CO_2_. Mice were then decapitated, 280-μm-thick coronal brain slices (280 mm) including the PAG region were prepared with a vibratome (Leica VT1200S) in the same ice-cold NMDG solution, and then maintained in this solution for 10 min at 34°C. After, they were transferred into the artificial CSF (ACSF; in mm: 126 NaCl, 2.5 KCl, 1.2 NaH_2_PO_4_, 1.2 MgCl_2_, 2.4 CaCl_2_, 11 glucose, and 25 NaHCO3, equilibrated with 95% O_2_ and 5% CO_2_) and kept at RT until use. For recording, slices were individually transferred to a chamber on an upright fluorescence microscope (Olympus BX51) and superfused continuously with ACSF (33°C, flow rate 2.5 ml min^−1^). PAG neurons were visualized with a 40× water‐immersion objective using Dodt gradient contrast optics and mCherry fluorescence was detected using epifluorescent illumination. Whole‐cell patch-clamp recordings were performed in the current-clamp configuration. Patch pipettes (3–4 MΩ) were filled with an intracellular solution composed of (in mm): 130 K-gluconate, 0.5 EGTA, 10 HEPES, 10 Na2-phosphocreatine, 5 MgATP, 0.4 NaGTP, and 0.1% biocytin, pH 7.3 with KOH (290–295 mOsm). The liquid junction potential was not corrected. Membrane potential was monitored in the presence of the AMPA/kainate receptor antagonist, 2,3-Dioxo-6-nitro-1,2,3,4-tetrahydrobenzo[f]quinoxaline-7-sulfonamide (NBQX; 5 μm), the NMDA receptor antagonist, d‐2‐Amino‐5‐phosphonopentanoic acid (D-AP5; 25 μm), and the GABA_A_ receptor antagonist picrotoxin (PTX; 100 μm). Loose cell-attached recordings were made using 2–3 MΩ glass pipettes containing normal ACSF at a holding potential at 0 mV in the voltage-clamp configuration under normal ACSF perfusion. Pipettes were gently pushed against the membrane of the mCherry-positive PAG cells (seal resistance: 5–20 MΩ). We used hM3Dq receptor-expressing cells which had <3-Hz basal frequency to avoid undetectable action potential (AP) currents after the application of CNO. A cell that had 8.8 Hz basal AP frequency reached >50 Hz 1 min after application of CNO. After this time, the AP amplitude became smaller and eventually stopped, presumably suggesting that in cells with a higher baseline AP frequency and a more depolarized membrane potential, that CNO-induced membrane depolarization and inactivates sodium channels. All recordings were filtered (2- to 10-kHz low pass filter) with Multiclamp 700B amplifier (Molecular Device) and digitized at a sampling rate of 10 kHz with an A/D converter (NI USB-6251, National Instruments), and stored using a data acquisition program (AxographX, Axograph Scientific Software). Off-line analysis was performed using Clampfit10 (Molecular Device) and Igor Pro 6 (WaveMetrics).

To visualize cells filled with biocytin during electrophysiology recording, brain slices were fixed in 10% formalin solution (Sigma) and then incubated with streptavidin Alexa-647 (1:1000, Abcam). Slices were rinsed in PBS, mounted with PermaFluor (Epredia), and stored at 4°C. The slices were imaged in a Leica TCS SP5 confocal microscope using a 40× (NA 1.25) objective.

### Drug application

All other drugs were bath applied: NBQX and D-AP5 were from Abcam. Picrotoxin was from Alomone. All other drugs were from Sigma-Aldrich. CNO was dissolved in DMSO for stock solutions (30 mm).

### Data analysis

The experimenter was blinded to the treatment group during data analysis. Statistical analyses were performed using GraphPad Prism (version 9, GraphPad Software). For c-Fos/mCherry colocalization the Student’s *t* test was used. For the nociception tests, one-way repeated measures (RM) ANOVA was used to consider interindividual variability within mice in different groups, followed by Bonferroni’s multiple comparison test. Normality was assessed by the Shapiro–Wilk test, and outliers were identified by the ROUT method (Q = 1%). If the data from one treatment per group were identified as an outlier, all the dataset for that mouse was removed from RM or Paired analysis. Data that was not normal was analyzed by Friedman ANOVA followed by Dunn’s multiple comparison test. Locomotor behavior was analyzed with ANY maze software (version 7.09), except for jumping and rearing behavior which was analyzed manually. Statistics were conducted using paired *t* tests to consider interindividual variability within mice in different groups. If the normality assumption was violated Wilcoxon signed rank test was used.

All data are presented as mean ± SEM and statistically significant results are indicated by an asterisk when *p* < 0.05.A detailed statistical summary can be found in Extended Data Table 1-1.

### Ethics statement

This work was carried out in accordance with the Australian code for the care and use of animals for scientific purposes, and assessed and approved by the Royal North Shore Animal Ethics committee. Genetically modified materials were used and disposed of with the approval of the Royal North Shore Hospital Institutional Biosafety committee.

## Results

### Chemogenetic targeting of GlyT2^+^ neurons in the PAG

The glycine transporter 2 (GlyT2) is expressed presynaptically in inhibitory neurons that use glycine as a neurotransmitter (McIntire et al., 1997; Sagné et al., 1997; Chaudhry et al., 1998; Dumoulin et al., 1999; Wojcik et al., 2006). Previous reports indicate that a population of GlyT2^+^ neurons are found in the midbrain PAG, and suggests that these neurons are concentrated in vlPAG (Rampon et al., 1996; Tanaka and Ezure, 2004; Zeilhofer et al., 2005), a region with well-established roles in descending modulation of pain and opioid-mediated analgesia (Behbehani, 1995; K.A. Keay and Bandler, 2015). To better understand the functional role of GlyT2-PAG neurons, we selectively expressed hM3Dq, hM4Di, or control (mCherry) protein in GlyT2-PAG neurons of male or female GlyT2::Cre mice (Foster et al., 2015). Then, we conducted behavioral testing using a within-subject crossover design to deliver the selective DREADD agonist clozapine N-oxide (CNO) or vehicle control (Fig. 1*A–C*).

**Figure 3.**
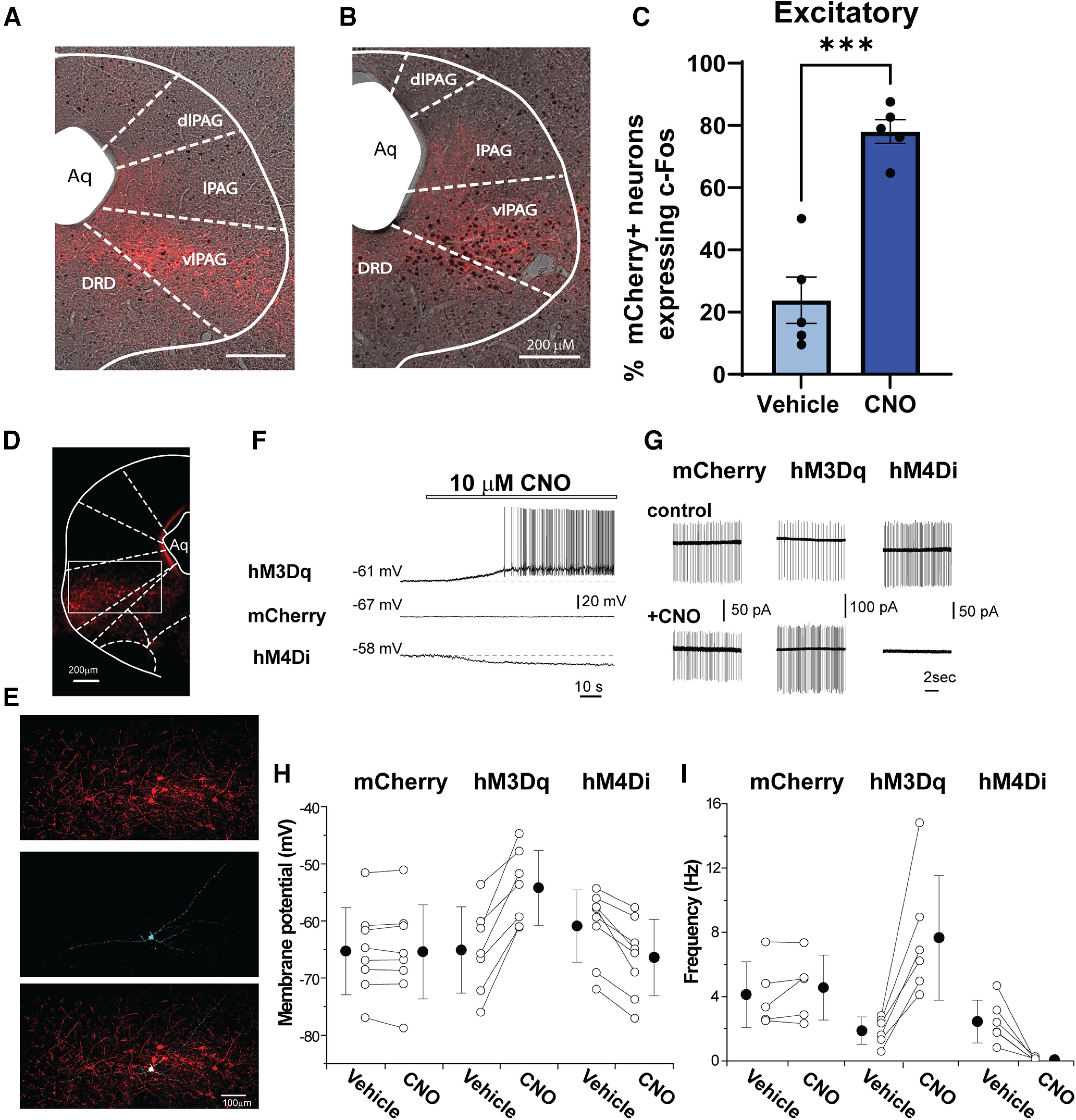
Functional characterization of hM3Dq and hM4Di in vlPAG neurons of GlyT2:Cre mice. ***A***, ***B***, c-Fos expression in GlyT2::cre neurons expressing hM3Dq was enhanced following CNO injection. Representative images of coronal PAG sections showing c-Fos (black spots) is strongly colocalized in DREADD-expressing neurons (mCherry^+^, red) following CNO (***B***, 3 mg/kg) compared with vehicle (***A***) injection (intraperitoneally). ***C***, Bar graph of the % mCherry^+^ neurons that were co-labeled with c-Fos following vehicle or CNO injection (24 ± 16.63% vs 78 ± 8.55%, *n* = 5). ***D***, Fluorescent image of vlPAG neurons expressing hM3Di-mCherry and a whole-cell recorded cell. ***E***, Enlarged images (mCherry, streptavidin, merged) of the square region in ***D***. ***F***, Examples of current-clamp recording from hM3Dq, mCherry and hM3Di-expressing vlPAG neurons during bath application of 10 μm CNO. Dashed lines indicated the membrane potential of the cells before CNO application. ***G***, Quantification of the CNO effect on membrane potential at 3 min after its application. ***H***, Examples of action potential currents from loose-cell attached recordings from mCherry, hM3Dq and hM3Di-expressing vlPAG neurons before and 5 min after 10 μm CNO application. ***I***, Quantification of the CNO effect on action potential frequency at 5 min after its application. Scale bars in ***D***, ***E***: 100 μm. *p < 0.05. All values are mean ± SEM. A control experiment in Cre-negative littermates is presented in Extended Data Figure 3-1.

Adeno-associated viral-vector (AAV5) mediated expression of transgenes in GlyT2::cre mice was strongly concentrated in the vlPAG (Fig. 2*A*,*B*), consistent with previous reports. To confirm that CNO activation of hM3Dq receptor enhanced the neuronal activity of GlyT2-PAG neurons, we combined IHC detection of c-Fos, an immediate early gene that is transiently expressed following neuronal activation (Perrin-Terrin et al., 2016), with IF labeling of hSyn-DIO-hM3Dq-mCherry neurons in mice that were injected with CNO (3 mg/kg, i.p.) or vehicle control 2 h before being killed. c-Fos was strongly colocalized hM3Dq-mCherry expressing vlPAG neurons following CNO-injected compared with vehicle-injected animals (CNO 78 ± 8.55%, vehicle 24 ± 16.63%, *p* = 0.0002, students *t* test, *n* = 5 animals per group; Fig. 3*A–C*).

10.1523/ENEURO.0069-23.2023.f3-1 Extended Data Figure 3-1 Control experiments in GlyT2:Cre negative mice confirm that AAV-Cre-independent leak of vectors did not contribute to the CNO-stimulated behavioral changes. AAV5-hSyn-DIO-hM3D(Gq)-mCherry was stereotaxically injected into the vlPAG of cre-negative littermates of GlyT2::cre mice. ***A***, Low-level cre-independent expression of mCherry was revealed following antibody amplification. Note: antibody amplification was not carried out in any of the injection site figures shown in the main text. ***B***, Leak expression had no functional effect as CNO (3 mg/kg, i.p.) administration did not alter hind paw licking, PWL or locomotion of cre-negative animals injected with hM3Dq (blue bars) compared to vehicle controls. *n* = 5, values are presented as mean ± SEM. Download Figure 3-1, TIF file.

In addition, we used electrophysiology to directly record DREADD-mediated neuronal modulation in coronal midbrain slices (Fig. 3*D*,*E*) from male and female mice in whole-cell current clamp (Fig. 3*F*) and loose patch configuration (Fig. 3*G*). We compared membrane potential and firing rates from hM3Dq/mCherry^+^, hM4Di/mCherry^+^ and mCherry^+^ control PAG neurons. Bath application of CNO (3–10 μm) had no significant effect on the membrane potential or firing rate in recordings from mCherry^+^ control neurons (control vs CNO: −66.0 ± 1.79 vs −66.3 ± 2.10 mV, *p* = 0.642; 3.25 ± 0.85 vs 3.56 ± 0.89 Hz, *p* = 0.249; two tailed paired *t* test, *n* = 7–8 cells; Fig. 3*F–I*). However, CNO significantly depolarized and increased the action potential firing rate of hM3Dq/mCherry^+^ expressing vlPAG neurons (control vs CNO: −65.1 ± 2.86 vs −54.2 ± 2.48 mV, *p* = 0.001; 1.87 ± 0.35 vs 7.66 ± 1.58 Hz, *p* = 008; two tailed paired *t* test, *n* = 6–7 cells; Fig. 3*F–I*) and significantly hyperpolarized and decreased the action potential firing of hM4Di/mCherry^+^ vlPAG neurons (control vs CNO: −60.9 ± 2.23 vs −66.4 ± 2.26 mV, *p* = 0.001; 2.44 ± 0.55 vs 0.06 ± 0.04 Hz, *p* = 0.008; two tailed paired *t* test, *n* = 6–7 cells; Fig. 3*F–I*). Together, these findings demonstrate that functional DREADDs are expressed in the vlPAG and that CNO increases and suppresses neuronal excitation in mice expressing hM3Dq and hM4Di respectively.

### Bidirectional control of acute nociceptive behaviors by GlyT2^+^ neurons in the vlPAG

**Figure 4.**
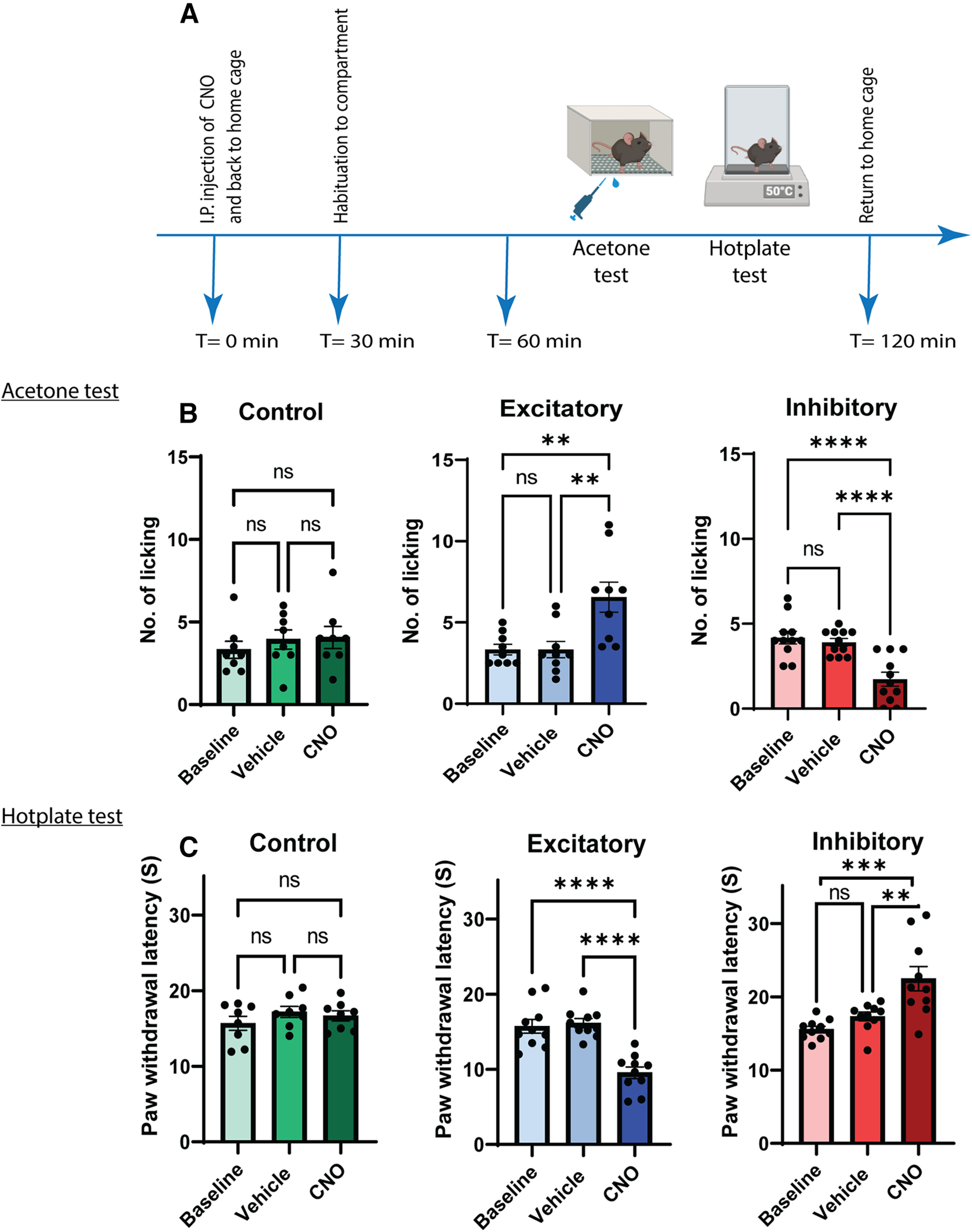
Chemogenetic manipulation of vlPAG GlyT2^+^ neurons bidirectionally modulates acute nociceptive behavior in male mice. ***A***, Schematic of the chemogenetic protocol for assessing acute nociceptive behaviors (acetone and hotplate) in male GlyT2::cre mice. ***B***, ***C***, CNO (3 mg/kg, i.p.) administration resulted in increased hind paw licking and decreased PWL in GlyT2::cre male animals injected with hM3Dq (blue bars). CNO (5 mg/kg, i.p.) administration resulted in decreased hind paw licking and increased PWL in GlyT2::cre animals injected with hM4Di (red bars) compared with vehicle injection. CNO (5 mg/kg, i.p.) administration had no effect on hind paw licking and PWL in GlyT2::cre animals injected with control mCherry vector (green bars). Individual animals are indicated on the graphs (*n* = 7–11) and values are presented as mean ± SEM, and significant results were determined when *p < 0.05.

To test the functional contribution of GlyT2-PAG neurons in acute pain behaviors, we conducted behavioral experiments three to four weeks after stereotaxic injection of AAV5 vectors encoding hM3Dq-mCherry, hM4Di-mCherry, or mCherry control mice (Fig. 4*A*).

Cold responses were measured using the acetone test. In male GlyT2::Cre mice expressing the excitatory DREADDs (hM3Dq; Fig. 4*B*, blue bars), CNO (3 mg/kg, i.p.) significantly increased the number of hind paw licking (No. licking) responses compared with vehicle injection (*F*_(2,16)_ = 10.03, *p* < 0.01, *n* = 9; Fig. 4*B*). In contrast, in GlyT2::Cre animals expressing the inhibitory DREADDs (hM4Di; Fig. 4*B*, red bars), CNO (5 mg/kg, i.p.) significantly decreased the No. licking compared with vehicle injection (*F*_(2,20)_ = 22.52, *p* < 0.0001, *n* = 11; Fig. 4*B*). CNO (5 mg/kg, i.p.) had no effect on No. licking in the mCherry control group (*F*_(2,14)_ = 0.54, *p*> 0.05, *n* = 8; Fig. 4*B*, green bars).

We also investigated the role of GlyT2^+^ vlPAG neurons on responses to noxious heat, using the hotplate test. In male GlyT2::Cre mice expressing hM3Dq (Fig. 4*C*, blue bars), CNO significantly decreased the paw withdrawal latency (PWL) to noxious thermal stimulation compared with vehicle injection (*F*_(2,18)_ = 30.85, *p* < 0.0001, *n* = 10; Fig. 4*C*). Whereas in the group expressing hM4Di (Fig. 4*C*, red bars), CNO significantly increased the PWL to noxious thermal stimuli compared with vehicle injection (*F*_(2,18)_ = 14.22, *p* ≤ 0.001, *n* = 10; Fig. 4*C*). CNO had no effect on the PWL in the mCherry control group (*F*_(2,14)_ = 1.47 *p*> 0.05, *n* = 8; Fig. 4*C*, green bars).

As vlPAG modulation of nociception has sex difference (Loyd et al., 2007; Loyd and Murphy, 2014; Rosen et al., 2017; Yu et al., 2021), we also conducted a parallel set of experiments on female GlyT2::Cre mice expressing either the excitatory or inhibitory DREADD under the same conditions. In female GlyT2::Cre mice expressing hM3Dq (Fig. 6*B*, blue bars), CNO significantly increased the No. licking responses compared with vehicle injection (*F*_(2,10)_ = 13.65, *p* < 0.01, *n* = 6; Fig. 6*B*) and significantly decreased the PWL to noxious thermal stimulation compared with vehicle injection (*F*_(2,10)_ = 10.91, *p* < 0.01, *n* = 6; Fig. 6*C*). In female GlyT2::Cre animals expressing hM4Di (red bars), CNO did not significantly decrease the No. licking compared with vehicle injection (*F*_(2,8)_ = 3.024, *p* > 0.05, *n* = 5; Fig. 6*B*) but significantly increased the PWL to noxious thermal stimuli compared with vehicle injection (*F*_(2,8)_ = 7.221, *p* < 0.05, *n* = 6; Fig. 6*C*). Together, these data indicate that the activity of GlyT2^+^ neurons in the vlPAG exerts bidirectional nociceptive control in both male and female mice.

**Figure 5.**
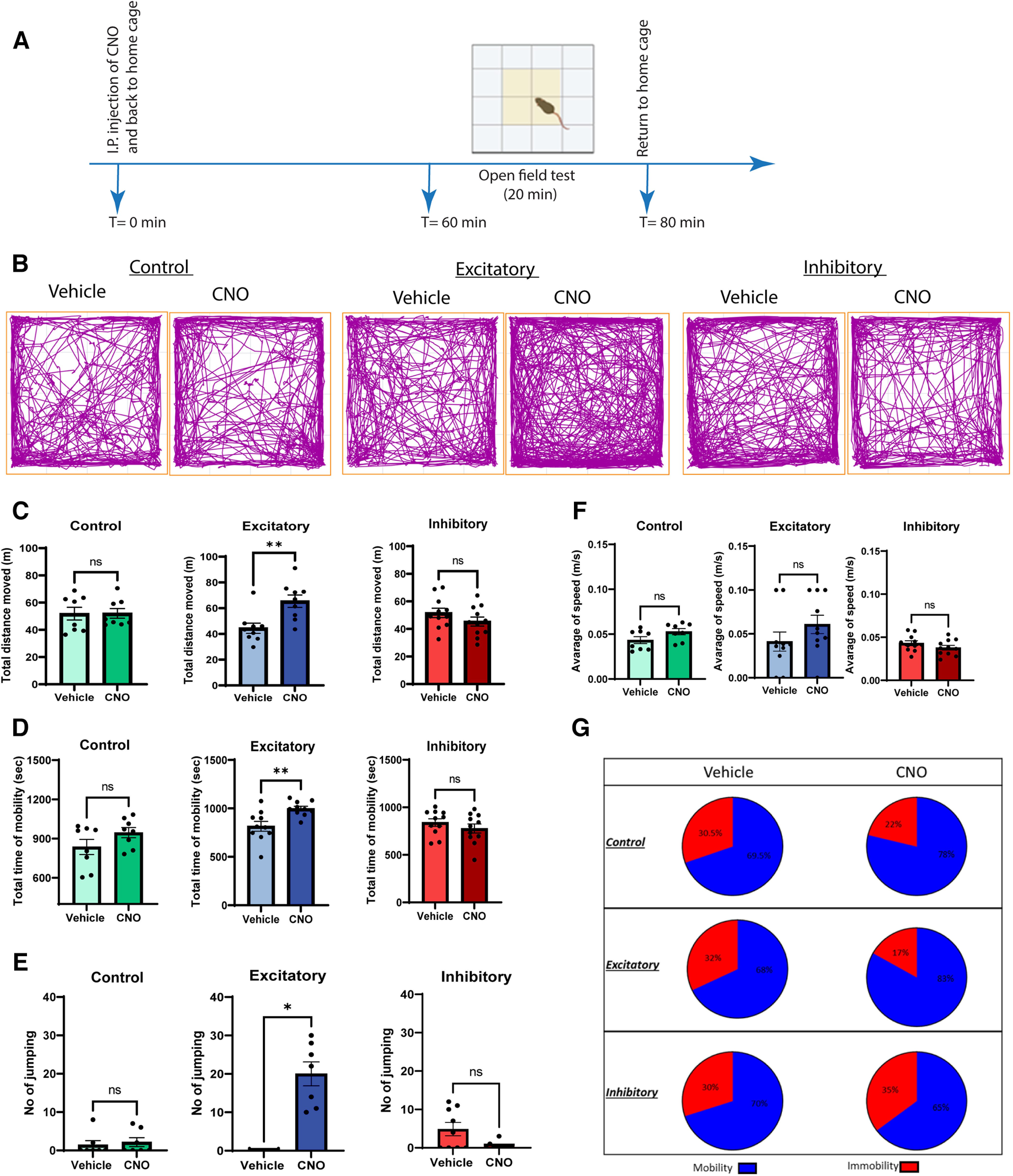
Chemogenetic activation of vlPAG GlyT2+ neurons alters locomotion behavior in the open field test in male mice. ***A***, Schematic of the chemogenetic protocol for assessing locomotor behaviors (open field) in male GlyT2::cre mice. ***B***, Plots show the position of the animals’ center point for the entire duration of the test. ***C–F***, CNO (3 mg/kg, i.p.) administration resulted in increased total distance moved, increased mobility time, and increased jumping behavior but did not alter the average speed of GlyT2::cre male animals injected with hM3Dq compared with vehicle (blue bars). In contrast, no significant differences were observed in locomotion behavior when CNO (5 mg/kg, i.p.) was administered to GlyT2::cre animals injected with hM4Di compared with vehicle (red bars). In addition, CNO (5 mg/kg, i.p.) had no effect on locomotion behaviors in GlyT2::cre animals injected with the control mCherry vector (green bars). ***G***, Pie charts representing the overall percentage changes in mobility versus immobility in both CNO and vehicle-injected animals expressing control (mCherry), excitatory (hM3Dq) and inhibitory (hM4Di) DREADDS. Individual animals are indicated on the graphs (*n* = 7–11) and values are presented as mean ± SEM and significant results were determined when *p < 0.05.

Finally, we assessed the locomotor effects of GlyT2^+^ vlPAG neuronal activity in the open field test over a 20 min test period (Fig. 5*A*). We scored behaviors including total distance traveled, average speed, im/mobility, rearing, jumping, line crossing, center zone entries and total time spent in the center zone.

CNO injection in male GlyT2::cre mice expressing hM3Dq (Fig. 5, blue bars) caused a significant increase in the total distance moved (*p* < 0.01, *n* = 9;Fig. 5*C*), total time of mobility (*p* < 0.01, *n* = 10; Fig. 5*D*), and an associated decrease in immobility time (*p* < 0.01, *n* = 10; Fig. 5*G*). In addition, the total number of line crossings (not shown, *p* < 0.05, *n* = 10), total number of center entries (not shown, *p* < 0.05, *n* = 6), and center zone distance traveled (not shown; *p* < 0.05, *n* = 6), were increased and a dramatic increase in jumping behavior (*p* < 0.05, *n* = 7; Fig. 5*E*) was observed. We did not detect a change in average speed (*p* > 0.05, *n* = 10; Fig. 5*F*), time spent in the center zone (not shown, *p* > 0.05, *n* = 9) or rearing behavior (not shown, *p* > 0.05, *n* = 9) compared with vehicle control.

**Figure 6.**
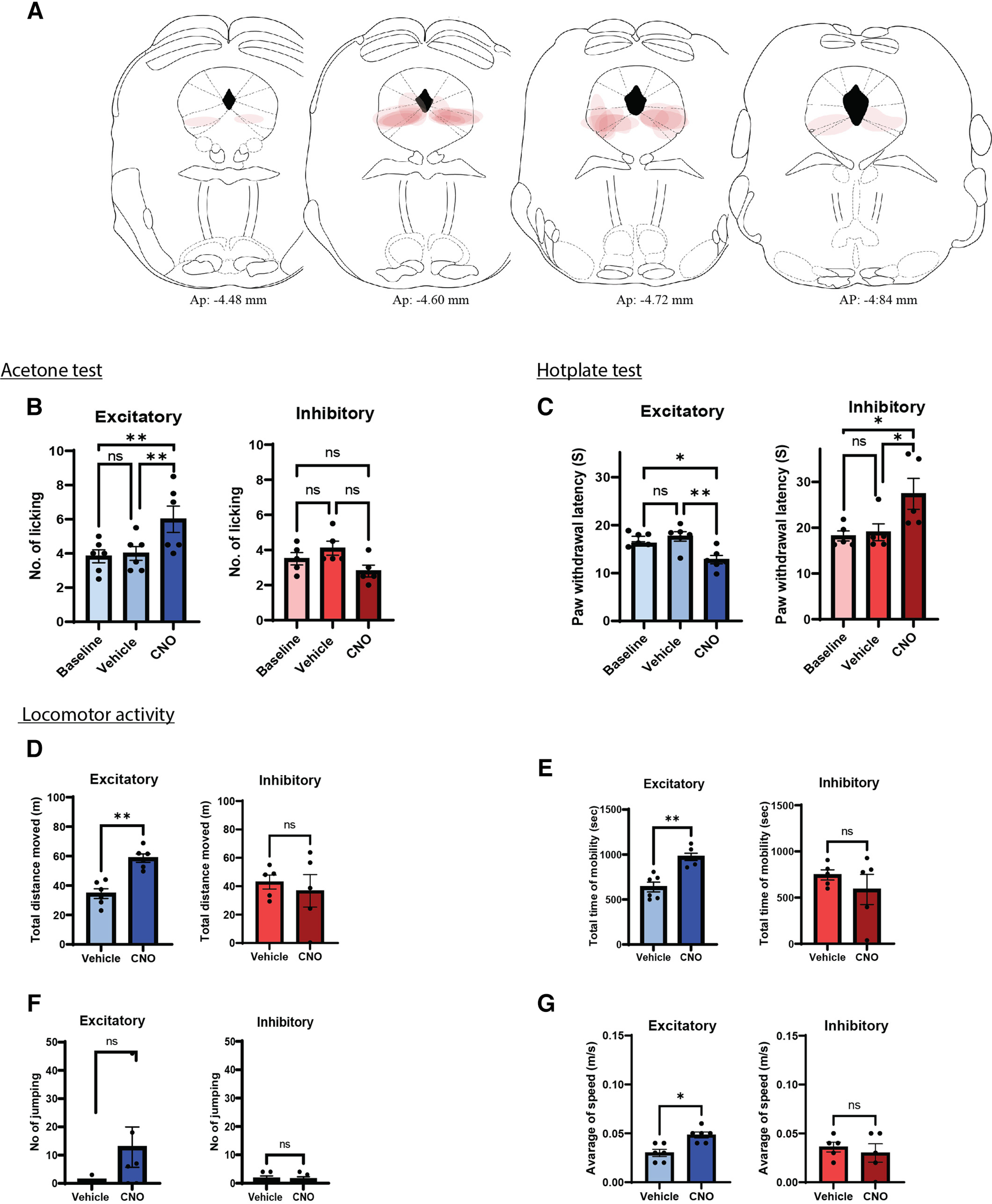
Chemogenetic manipulation of vlPAG GlyT2^+^ neurons has bidirectional effects on nociception and increases locomotion in female mice. Female GlyT2::cre mice were tested with the same protocols as shown in Figures 4, 5. ***A***, Placement map of AAV5 viral vectors expression in vlPAG with the injection represented at 10% opacity for each animal. Bar graphs showing the responses to acetone (***B***) and hotplate (***C***) of mice expressing either excitatory (hM3Dq) or inhibitory (hM4Di) DREADDs and following injection with either vehicle or CNO (3–5 mg/kg, i.p., respectively) ***D–G***, Bar graphs of relevant locomotion parameters: total distance (***D***), total time of mobility (***E***), jumping (***F*)** and average speed (***G***). Individual animals are indicated on the graphs (*n* = 5–6). Values are presented as mean ± SEM and *p < 0.05.

Comparable locomotor results were measured following CNO injection in hM3Dq expressing female mice (Fig. 6, blue bars), with the exception of jumping behavior, which was not significantly altered (*p* > 0.05, *n* = 5; Fig. 6*F*) and average speed, which increased (*p* < 0.05, *n* = 6; Fig. 6*G*), a finding that is consistent with a previous report (Capelli et al., 2017).

No alteration in locomotor behaviors were detected in hM4Di expressing male or female mice [male: *p* > 0.05, *n* = 10–11 (Fig. 5*C–F*, red bars); female: *p* > 0.05, *n* = 5–6 (Fig. 6*D–G*, red bars)] or in mCherry control mice (*p* > 0.05, *n* = 7–8; Fig. 5*C–F*, green bars) injected with CNO compared with their vehicle controls (*p* > 0.05, *n* = 7–11; Fig. 5*C–F*). These data suggest that activation, but not inhibition of GlyT2^+^ vlPAG neurons exerts a stimulatory effect on exploratory locomotor behaviors in both male and female mice.

## Discussion

### GlyT2+ neurons have a crucial role in pain modulation

The GlyT2::Cre mouse allows us to selectively manipulate a sparse subset of neurons concentrated primarily in the ventrolateral subdivision of the PAG and to investigate their functional role. We demonstrate that the activity of GlyT2-PAG neurons bidirectionally modulate acute nociceptive responses, and that their activation increases locomotion. These findings can be directly compared with a recent study that used viral vectors to express chemogenetic channels in the vlPAG of male vGAT-Cre mice (Samineni et al., 2017) and monitored nociceptive (but not any other) behaviors. Consistent with our findings, activation of vGAT-PAG neurons was pronociceptive and inhibition was antinociceptive. As the vesicular transporter vGAT is responsible for concentrating both GAD-synthesized GABA and GlyT2-supplied glycine into synaptic vesicles (Burger et al., 1991; McIntire et al., 1997; Wojcik et al., 2006; Aubrey et al., 2007), and because GlyT2 has been shown to be strongly colocalized with GABAergic neurons in the PAG (Tanaka and Ezure, 2004; Vaughn et al., 2022), we consider that the GlyT2-PAG population are a minority subset of all the inhibitory neurons manipulated by Samineni et al. (2017). Thus, our study demonstrates that the activity of a small population of inhibitory neurons, whose cell bodies are confined within the vlPAG (Figs. 2, 6; Rampon et al., 1996; Zeilhofer et al., 2005), can effectively control acute nociceptive responses. Further, as GlyT2-PAG neurons bidirectionally modulate acute nociceptive responses, their baseline activity likely contributes to setting heat and cold sensitivity thresholds. Interestingly, we found that despite their relative scarcity, the scale of the nociception change achieved by manipulating GlyT2-PAG neurons was similar to that reported by Samineni et al. (2017). For example, we report that inhibition of GlyT2-PAG neurons increased PWL (noxious heat) by 130.2%, whereas inhibition of vGAT-PAG neurons led to a 136.3% increase in PWL. Given the relative density of GlyT2 neurons is small (Tanaka and Ezure, 2004; Vaughn et al., 2022), this observation could be explained if GlyT2-PAG neurons exert a more targeted control of the descending pain modulatory pathways than the vGAT^+^ population, or that the net behavioral output measured following manipulation of all vlPAG vGAT^+^ neurons reflect a composite behavioral output that is not maximal. In any case, these findings demonstrate that GlyT2-PAG neurons can robustly modulate acute nociception and suggests that treatments designed to selectively inhibit GlyT2-PAG neurons may be effective analgesics. An advantage of this type of modulation is that it would preserve GABAergic inhibitory function in the PAG and indeed in cortical regions, as there are no GlyT2^+^ neurons in the cortex (Rampon et al., 1996; Zeilhofer et al., 2005). Interestingly, we were also able to detect significant changes in nociceptive responses in a small group of animals that received a unilateral injection of hM3Dq [data not shown; *p* = 0.02 (acetone), *p* = 0.04 (hotplate), *n* = 5], suggesting partial engagement of this neuronal population is sufficient to alter behavior.

### Interpretation of locomotor behaviors

The data show that activation of GlyT2-PAG neurons increased locomotor activity in male and female mice. The open-field test allows a systematic assessment of rodent exploration and general locomotor behavior that can be used for the initial screening of anxiety-like behaviors; as the open-field environment is a novel, exposed, white-lit space that temporarily isolates the animal from its cage mates (Bailey and Crawley, 2009). In response to stress, animals generally employ two main coping strategies that depend to a large extent on their state and the environmental context. Either an active (increases in mobility behavior, running or jumping behavior) or a passive (increases in immobility or freezing) fear response. Our finding that chemogenetic activation of GlyT2-PAG neurons expressing hM3Dq increased total distance moved, mobility and jumping behavior in male mice, and total distance moved, mobility and speed in female mice, are consistent with active fear-coping responses that are beyond exploratory behavior. This suggests that GlyT2-PAG neurons may influence neuronal circuits involved in both nociception and anxiety-like behaviors. We did not observe any changes in the time spent in the center zone, which has been associated with increased anxiety/stress in mice and increased locomotor activity in escapable environments (Korte et al., 1999). However, mobility and center zone times are not always correlated, and conclusively determining a role for GlyT2-PAG neurons in anxiety requires more specific tests such as the elevated plus maze and the light dark test (Korte et al., 1999; Bourin and Hascoët, 2003) .

### The vlPAG contains several distinct effector circuits

In addition to the well-described descending pain modulatory pathway which connects the vlPAG to the dorsal horn of the spinal cord via the RVM, the vlPAG houses other descending and ascending projection neurons that are involved in coordinating a host of other threat responses (Cameron et al., 1995b; K.A. Keay and Bandler, 2001). Recent studies have started to unravel the organization and describe distinct functions for these circuits (De Luca-Vinhas et al., 2006; Tovote et al., 2015; Koutsikou et al., 2017). For instance, both PAG-CCK expressing glutamatergic PAG neurons and low-dose NMDA injections influence different aspects of defensive behaviors and nociception (de Mello Rosa et al., 2022; La-Vu et al., 2022). On the other hand, vlPAG neurons that project to the medullary magnocellular nucleus (Mc) are responsible for freezing, locomotion, and coordinating defensive responses, but do not affect thermal nociception (Tovote et al., 2016; Capelli et al., 2017). Our research showing that activation of GlyT2-PAG neurons increases locomotion, aligns with the proposed function of the vlPAG-Mc pathway. However, GlyT2-PAG inhibition did not significantly reduce locomotion as expected and their activation is also linked with increased nociceptive behaviors, suggesting that another independent pathway may be involved.

### Glyt2-PAG neurons modulate nociception and locomotion in male and female mice

It is clear that the anatomy and physiology of pain modulation in male and female animals are distinctive (Loyd and Murphy, 2014; Rosen et al., 2017), and that signaling in the vlPAG contributes to these differences (Loyd et al., 2007; Capelli et al., 2017; Doyle et al., 2017; Yu et al., 2021; Llorente-Berzal et al., 2022). We conducted experiments in both male and female mice and found that GlyT2-PAG neurons similarly modulate the nociceptive and locomotive behaviors tested. However, we found that the quality of locomotor response triggered by GlyT2-PAG neuronal activation was qualitatively different between the sexes, with the increased mobility observed accompanied by jumping behaviors in male mice, and increased speed in female mice. These findings support a role for GlyT2-PAG neurons in modulating pain responses in both sexes and highlight the need to carefully consider and report appropriate behavioral outcomes for mice of different sexes.

### Technical considerations

The chemogenetic DREADDs approach provides a powerful and precise technique to selectively manipulate a particular subpopulation of neurons in neural circuits. The advantage of this method is that it gives researchers the possibility to modulate specific neuronal populations in isolation and observe the resultant behavioral consequences. However, chemogenetic modulation of circuits is not physiological, as it depolarizes or hyperpolarizes target neurons over a sustained period (a few hours), and the effect of prolonged DREADD activation on neuronal firing will be determined by each neuron type, dependent on the types of channels it expresses and its repolarisation capability (Smith et al., 2016). Experiments using optogenetics to achieve a faster and more phasic engagement of these cells will mitigate this problem and could potentially reveal a more nuanced view of GlyT2-PAG modulation of nociception and locomotion. Another advantage of optogenetics is that specific projection pathways can be targeted using precise stereotaxic injection of the viral vector expressing opsin and placement of the optic fiber or intersectional viral approaches strategies. As with all techniques that rely on viral vectors and genetic technologies, alterations in circuit organization, synaptic transmission properties and axonal morphology because of the use of vector-mediated protein expression and foreign protein expression/activation must also be considered (Miyashita et al., 2013; Jackman et al., 2014; Owen et al., 2019).

CNO can have off-target biological effects (Goutaudier et al., 2019). To minimize this possibility, we have used low-medium doses of CNO, which do not cause demonstrable off-target behavioral activity in control DIO-mCherry mice (MacLaren et al., 2016; Gomez et al., 2017; Bærentzen et al., 2019). Furthermore, in our electrophysiological experiments, changes in the membrane potential and firing rate of vlPAG neurons expressing hM3Dq and hM4Dq were always recorded in response to CNO application, whereas no change was detected in mCherry-expressing midbrain neurons. Finally, in cre-negative littermates of GlyT2::cre mice injected with AAV-DIO- hM3Dq vectors, CNO had no measurable behavioral effect. For these reasons, we are confident the reported effects are correlated with the expected changes in GlyT2-PAG activity.

### Future directions

Future studies should investigate how and with whom GlyT2-PAG neurons communicate. Although the behavioral outcomes of altering GlyT2-PAG neuron activity are consistent with them being a subpopulation of inhibitory GABAergic neurons, their potential to release glycine means that their signaling mechanisms are likely to be distinct. Within the PAG, glycine could alter network activity by interacting with inhibitory glycine receptors or excitatory NMDA receptors (Ahmadi et al., 2003; Lynch, 2009).

A possible role for postsynaptic glycine receptors is suggested by experiments using electrophysiology to record evoked IPSCs in the ventrolateral PAG, where inhibitory currents with a glycine receptor-mediated component are occasionally detected. Indeed, this is the reason for the inclusion of strychnine (the specific glycine receptor antagonist) in slice recordings of the PAG (Tonsfeldt et al., 2016; Aubrey et al., 2017; Winters et al., 2022; Natale et al., 2023). In addition, glycine may be stimulating PAG activity. First, glycine activation of presynaptic glycine receptors expressed on glutamate-releasing neurons has been shown to increase the frequency of spontaneous EPSCs in acutely dissociated PAG neurons (Choi et al., 2013). Second, the modulation of nociceptive behavior by intra-PAG microinjections of glycine is sensitive to the glycine-site NMDA receptor antagonist 7-Cl-kynurenic acid (Palazzo et al., 2009). Determining the signaling mechanism employed by GlyT2-PAG neurons with in the PAG will permit a more complete understanding of how GlyT2-PAG neurons fit into the PAG circuitry.

In conclusion, this study demonstrates that GlyT2-PAG neurons effectively and bidirectionally modulate nociceptive responses in mice and indicates they may also play a role in anxiety-like behaviors. Until recently, our understanding of vlPAG function has been primarily gleaned from global manipulations of all or most of the neurons in the area. To better understand PAG function and nociceptive control, there is a need to carefully investigate the function of subpopulations of neurons in the vlPAG and to identify which microcircuits within the PAG are active under a range of threatening situations (Yin et al., 2020; Yang et al., 2022). These types of studies will continue to improve the functional frameworks used to understand how this region coordinates dynamic nociceptive responses and give insights into how common comorbidities develop in chronic pain states.

10.1523/ENEURO.0069-23.2023.t1-1 Extended Data Table 1-1 **Detailed statistical data for all figures** Download Table 1-1, XLS file.

## Synthesis

Reviewing Editor: Nathalie Ginovart, Universite de Geneve Decisions are customarily a result of the Reviewing Editor and the peer reviewers coming together and discussing their recommendations until a consensus is reached. When revisions are invited, a fact-based synthesis statement explaining their decision and outlining what is needed to prepare a revision will be listed below. The following reviewer(s) agreed to reveal their identity: Susan Ingram.

The reviewers expressed interest in your manuscript and agreed on the significance of the work as a novel contribution to the field. A few minor points, however, should be addressed in a review to improve the clarity of the manuscript.

Please find the specific comments raised by the reviewers below:

Reviewer 1 :

The authors explore the role of neurons that contain Gly2T in the vlPAG in behavior using a chemogenetic approach. OVerall, this is well done with appropriate discussion of key issues and caveats. I really only had 2 issues that I think need to addressed.

1) the authors perform hotplate after the acetone assay...is this common, might this impact the results. I dont think it needs to be redone, but some discussion of this point is warranted.

2) the authors posit that glycine may play a more tonic role on regulation of function. There is no data demonstrating this, in fact glycine is a funny neurotransmitter as it can activate GlyR but also NMDA. It would be great if the authors could show how activation of these neurons impacts other neurons in the PAG, but that may not be possible. in the absence of that, some discussion of this issue is warranted.

Reviewer 2 :

The manuscript “Bidirectional modulation of nociception by GlyT2+ neurons in the ventrolateral periaqueductal gray” examines the role of a small population of glycinergic neurons in the ventrolateral periaqueductal gray (vlPAG) in modulating thermal pain thresholds and locomotor activity. The studies use adult male and female transgenic mice with Cre-recombinase under the glycine transporter (GlyT2) promoter and stereotaxic injections of viral excitatory and inhibitory DREADDs to selectively activate and inhibit the GlyT2+ neurons, respectively. Control experiments with Cre-negative littermates determined that there were no behavioral effects of Cre-leakage. Acute cold (acetone test) and hot (hotplate) stimuli were used to test for changes in pain threshold. Important controls to confirm that the excitatory DREADD produced excitation and inhibitory DREADD produced inhibition of GlyT2 neurons in the vlPAG with electrophysiology and FOS immunohistochemistry were performed. Activation of the GlyT2 neurons with CNO resulted in enhanced nociceptive behaviors in both thermal tests, whereas inhibition resulted in antinociceptive behaviors. There were no differences between males and females. Locomotor activity was increased with activation of the GlyT2 neurons, also in both sexes with some differences in the quality of the movement. Inhibition of the GlyT2 neurons had no behavioral effects in either sex. The data recapitulate effects on nociceptive thresholds in a recent publication examining DREADD control of GABAergic neurons and interestingly show that activating a smaller population of inhibitory neurons, known to coexpress GABA, has the same magnitude of effects. The results suggest that the typically used markers for GABA versus glutamate are not particularly informative in the PAG as there are multiple subpopulations of neurons that contain these neurotransmitters. Therefore, this study, and the interpretations of the results from the study, are important contributions to the field. In addition, the results suggest that glycinergic signaling may be a useful target for novel therapeutic strategies for pain.

Overall, the data in this manuscript are clearly displayed and carefully executed. The experimental design are statistics are provided and appropriate. There is one section in the Discussion (page 22-23, “The vlPAG contains several distinct effector circuits”), that is more dense with several run-on sentences that could be simplified to be more clearly presented.

## Author Response

Comments from Reviewer 1

Comment 1: the authors perform hotplate after the acetone assay...is this common, might this impact the results. I dont think it needs to be redone, but some discussion of this point is warranted.

Response: It is common for pain laboratories within our Institute and internationally to test thermal nociceptive assays in conjunction with other nociceptive assays on the same day. Using this method, we observed consistent data with single compared to multiple testing regimes. Importantly, both tests are transient - with stimulus and responses completed in 20-45 seconds and each test is carried out with > 5 mins recovery between each stimulus to maintain independent results.

We have reinforced this point by modifying the text and adding references in the methods section as follows: “Acute nociception was evaluated by measuring responses to cold (acetone) and hot (hotplate) stimuli applied to the left hind paw (> 5 minutes between tests to ensure results are independent (Anderson et al., 2014; Francois et al., 2017; Samineni et al., 2017b; Mitchell et al., 2021)).” Comment 2: the authors posit that glycine may play a more tonic role on regulation of function. There is no data demonstrating this, in fact glycine is a funny neurotransmitter as it can activate GlyR but also NMDA. It would be great if the authors could show how activation of these neurons impacts other neurons in the PAG, but that may not be possible. in the absence of that, some discussion of this issue is warranted

Response: In the discussion text we suggest GlyT2-PAG neurons may “exert a more targeted tonic control of the descending pain modulatory pathways than the vGAT+ population”. Our finding that chemogenetic manipulations result in a bidirectional change in thermal response suggests that GlyT2-PAG neurons have a tonic level of activity that helps set thermal thresholds. Eg. Inhibiting neurons that have no baseline activity would not be expected to have any effect on behaviour.

To better link this idea to the data presented, the term tonic has been removed from the current discussion text, and the following sentence added to the earlier paragraph in the discussion: “Thus, our study demonstrates that the activity of a small population of inhibitory neurons, whose cell bodies are confined within the vlPAG (Fig. 2, 6 & (Rampon et al., 1996; Zeilhofer et al., 2005)), can effectively control acute nociceptive responses. Further, as GlyT2-PAG neurons bidirectionally modulate acute nociceptive responses, their baseline activity likely contributes to setting heat and cold sensitivity thresholds.”

We agree with the reviewer that glycine has a few potential signalling partners in the PAG including NMDA receptors and that understanding how our neurons signal in the PAG is of interest. We have previously recorded evoked inhibitory postsynaptic currents in the ventrolateral PAG and very occasionally we record glycine receptor-mediated postsynaptic currents which are sensitive to the specific antagonist strychnine. For this reason, we and others often include strychnine in electrophysiological experiments in PAG slices. In addition, glutamate release may be modulated by presynaptic glycine receptors in the PAG and an early paper investigating the effects of glycine microinjection in to the PAG, suggests glycine’s interaction with NMDA receptors may modulate nociceptive behaviour. This has now been outlined with references in a new section of the discussion: “Future directions. Future studies should investigate how and with whom GlyT2-PAG neurons communicate. Although the behavioural outcomes of altering GlyT2-PAG neuron activity are consistent with them being a subpopulation of inhibitory GABAergic neurons, their potential to release glycine means that their signaling mechanisms are likely to be distinct. Within the PAG, glycine could alter network activity by interacting with inhibitory glycine receptors or excitatory NMDA receptors (Ahmadi et al., 2003; Lynch, 2009).

A possible role for postsynaptic glycine receptors is suggested by experiments using electrophysiology to record evoked inhibitory postsynaptic currents in the ventrolateral PAG, where inhibitory currents with a glycine receptor-mediated component are occasionally detected. Indeed, this is the reason for the inclusion of strychnine (the specific glycine receptor antagonist) in slice recordings of the PAG (Tonsfeldt et al., 2016; Aubrey et al., 2017; Winters et al., 2022; Natale et al., 2023). In addition, glycine may be stimulating PAG activity. First, glycine activation of presynaptic glycine receptors expressed on glutamate-releasing neurons has been shown to increase the frequency of spontaneous excitatory postsynaptic currents in acutely dissociated PAG neurons (Choi et al., 2013). Secondly, the modulation of nociceptive behaviour by intra-PAG microinjections of glycine is sensitive to the glycine-site NMDA receptor antagonist 7-Cl-kynurenic acid (Palazzo et al., 2009). Determining the signaling mechanism employed by GlyT2-PAG neurons with in the PAG will permit a more complete understanding of how GlyT2-PAG neurons fit into the PAG circuitry.”

Comments from Reviewer 2

Comment 1: There is one section in the Discussion (page 22-23, “The vlPAG contains several distinct effector circuits”), that is more dense with several run-on sentences that could be simplified to be more clearly presented.

Response: Thank you for your careful reading of the manuscript. We have reformulated this section of the manuscript to improve its clarity as follows: “In addition to the well-described descending pain modulatory pathway which connects the vlPAG to the dorsal horn of the spinal cord via the RVM, the vlPAG houses other descending and ascending projection neurons that are involved in coordinating a host of other threat responses (Cameron et al., 1995b; Keay and Bandler, 2001). Recent studies have started to unravel the organization and describe distinct functions for these circuits (De Luca-Vinhas et al., 2006; Tovote et al., 2015; Koutsikou et al., 2017). For instance, both PAG-CCK expressing glutamatergic PAG neurons and low-dose N-methyl-D-aspartic acid (NMDA) injections influence different aspects of defensive behaviours and nociception (de Mello Rosa et al., 2022 ; La-Vu et al., 2022). On the other hand, vlPAG neurons that project to the medullary magnocellular nucleus (Mc) are responsible for freezing, locomotion, and coordinating defensive responses, but do not affect thermal nociception (Tovote et al., 2016; Capelli et al., 2017). Our research showing that activation of GlyT2-PAG neurons increases locomotion, aligns with the proposed function of the vlPAG-Mc pathway.

However, GlyT2-PAG activation is also linked with increased nociceptive behaviours, and their inhibition did not significantly reduce locomotion as expected, suggesting that another independent pathway may be involved.”
