# Supplementary figures and images for: Bidirectional Modulation of Nociception by GlyT2+ Neurons in the Ventrolateral Periaqueductal Gray
Source: eNeuro. 2023 Jun 12;10(6):ENEURO.0069-23.2023. doi: 10.1523/ENEURO.0069-23.2023 (PMC10270318; doi:10.1523/ENEURO.0069-23.2023)

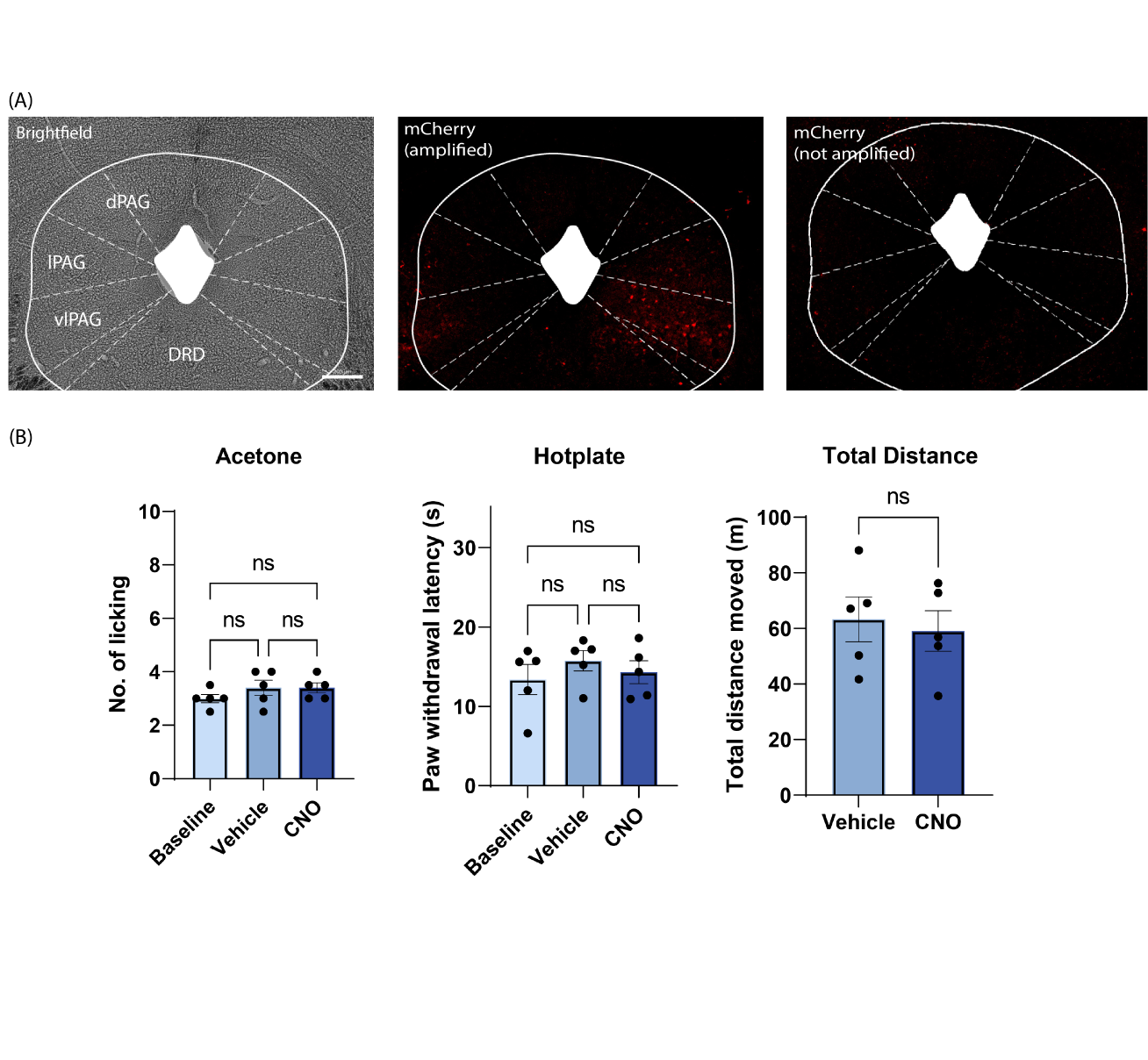

Supplement: Extended Data Figure 3-1 — Control experiments in GlyT2:Cre negative mice confirm that AAV-Cre-independent leak of vectors did not contribute to the CNO-stimulated behavioral changes. AAV5-hSyn-DIO-hM3D(Gq)-mCherry was stereotaxically injected into the vlPAG of cre-negative littermates of GlyT2::cre mice. A, Low-level cre-independent expression of mCherry was revealed following antibody amplification. Note: antibody amplification was not carried out in any of the injection site figures shown in the main text. B, Leak expression had no functional effect as CNO (3 mg/kg, i.p.) administration did not alter hind paw licking, PWL or locomotion of cre-negative animals injected with hM3Dq (blue bars) compared to vehicle controls. n = 5, values are presented as mean ± SEM. Download Figure 3-1, TIF file. [file enu-eN-NWR-0069-23-s02.tif]
